# Liver fibrosis: a compilation on the biomarkers status and their significance during disease progression

**DOI:** 10.4155/fsoa-2017-0083

**Published:** 2017-10-05

**Authors:** Krishna Sumanth Nallagangula, Shashidhar Kurpad Nagaraj, Lakshmaiah Venkataswamy, Muninarayana Chandrappa

**Affiliations:** 1 Department of Biochemistry, Sri Devaraj Urs Medical College, SDUAHER, Tamaka, Kolar, Karnataka, India; 2 Department of Medicine, Sri Devaraj Urs MedicalCollege, SDUAHER, Tamaka, Kolar, Karnataka, India; 3 Department of Community Medicine, Sri DevarajUrs Medical College, SDUAHER, Tamaka, Kolar, Karnataka, India

**Keywords:** biomarker, biomarker discovery, genetic markers, hepatic regeneration, liver fibrosis, -omics, sensitivity and specificity, validation strategies

## Abstract

Liver fibrosis occurs in response to different etiologies of chronic liver injury. Diagnosing degree of liver fibrosis is a crucial step in evaluation of severity of the disease. An invasive liver biopsy is the gold standard method associated with pain and complications. Biomarkers to detect liver fibrosis include direct markers of extracellular matrix turnover and indirect markers as a reflection of liver dysfunction. Although a single marker may not be useful for successful management, a mathematical equation combining tests might be effective. The main purpose of this review is to understand the diagnostic accuracy of biomarkers and scoring systems for liver fibrosis. Advances in -omics approach have generated clinically significant biomarker candidates for liver fibrosis that need further evaluation.

## Lay abstract

Liver fibrosis is a global health issue caused by various factors. Early diagnosis of the disease is important for better patient care. Liver biopsy is one of the diagnostic tools but comes with complications. Direct (involved in disease progression) and indirect markers are indicators for liver dysfunction that have easy applicability with less diagnostic value. Combination of these markers may give significant diagnosis but early detection is uncertain. Hence, the present review explains existing biomarkers and their relevance for the generation of ideal biomarkers for effective disease management by using advanced technology.

Liver fibrosis is a natural wound healing response which results in the formation of abnormal continuation of connective tissue production and deposition in response to chronic liver injury [1]. Causes of liver fibrosis are multifactorial and include congenital, metabolic, inflammation and toxins. In all these circumstances, replacement of parenchyma by fibrotic tissue, regenerative nodule and loss of liver functions are common [2]. Recent studies to understand the process of hepatic fibrogenesis show that treatment aimed at the underlying cause especially in earlier stage of the disease may improve or even reverse fibrosis. Reasons for resolution may be due to increase in collagenolytic activity and/or increased matrix metalloproteinase (MMP) activity due to decrease in expression of tissue inhibitor of metalloproteinase I (TIMP-I) [3]. Studies have reported that cytokine mobilization of bone marrow derived stem cells will restore neutrophil function and promote hepatic regeneration [4].

In normal liver, extracellular matrix (ECM) is present in space of Disse in direct contact with low-density basal lamina with glycoproteins, proteoglycans and glycosaminoglycans. After an acute liver injury, necrotic or apoptotic cells will be replaced by regenerated parenchymal cells. If the hepatic injury is chronic, there will be failure of regeneration and substitution of hepatocytes with abundant ECM and fibrillar collagen (Figure 1) [5]. Liver fibrosis is associated with major alterations in both quantity and composition of ECM. In advanced stage, fibrotic liver contains three- to ten-times more ECM than normal liver which includes collagens (I, III and IV), fibronectin, elastin, laminin, hyaluronic acid (HA) and proteoglycans [6].

**Figure 1.**
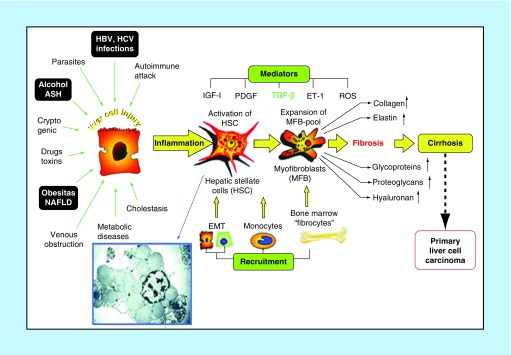
**Pathophysiology of liver fibrosis.** After chronic liver injury, necrotic or apoptotic cells will be replaced by regenerated parenchymal cells. Inflammation-connected activation of hepatic stellate cells takes place and transdifferentiation into myofibroblast-like cells which attains contractile, proinflammatory and fibrogenic property. ASH: Alcoholic steatohepatitis; EMT: Epithelial mesenchymal transition; ET-1: Endothelin-1; HSC: Hepatic stellate cell; NAFLD: Nonalcoholic fatty liver disease; ROS: Reactive oxygen species. Reproduced with permission from [5].

ECM-producing cells in the injured liver are hepatic stellate cells (HSC) which dwell in the space of Disse and are the major storage cells of vitamin A [7]. Due to chronic liver injury, activation of HSCs takes place and transdifferentiate into myofibroblast-like cells and attains contractile, proinflammatory and fibrogenic property. Chief mitogen for activation of HSCs is PDGF which is produced by Kupffer cells. Activated HSCs migrate and accumulate at tissue repair sites and secrete large amounts of ECM and regulates ECM degradation. HSCs collagen synthesis is regulated at transcription and post-transcriptional levels [7]. Replacement of normal low-density matrix by high-density interstitial matrix disturbs the hepatocyte synthetic and metabolic function and impairs solute transport from sinusoid to hepatocyte. Cellular behavior alterations are mediated by cell membrane receptors termed as integrins [6,8].

The activation of HSCs takes place in two phases (initiation and perpetuation). Initiation includes early changes in HSCs resulting from paracrine stimuli by neighboring cells viz., sinusoidal endothelium, kupffer cells, hepatocytes and platelets. Inflammatory marker cells stimulate matrix synthesis, cell proliferation and release of vitamin A by HSC through the action of cytokine TGF-β, reactive oxygen intermediates and lipid peroxides. Perpetuation involves seven discrete changes in cell behavior; proliferation, chemotaxis, fibrogenesis, contractility, matrix degradation, retinoid loss and inflammatory signaling, and WBC chemoattraction with cytokine release. Among the discrete changes in cell behavior following the perpetuation of HSC activation, fibrogenetic factors play a vital role in fibrogenesis [6,8].

Though the liver biopsy is considered the gold standard method, it is an invasive procedure associated with pain and complications. Scoring system for diagnosis and prognosis of fibrosis include routine laboratory tests viz., serum proteins, liver enzymes, bilirubin, prothrombin time (PT) and direct markers of ECM turn over. Liver fibrosis can be diagnosed by imaging techniques such as ultrasonography, computed tomography and MRI [7]. These diagnostic modalities can detect parenchymal changes but required skills and costs are exorbitant. In the present review, an attempt has been made to understand the diagnostic accuracy, advantages and disadvantages (Box 1) for existing biomarkers and different scores for liver fibrosis.

**Box 1.**  **Advantages and disadvantages of biomarkers of liver fibrosis.**

**Advantages:**

- Minimal invasive
- Not associated with morbidity and mortality
- Easy to apply with great availability and easier reproducibility
- Validated biomarkers with scores may be useful for monitoring therapy
- Biomarkers are less expensive

**Disadvantages:**

- Direct markers are not organ specific, influenced by unrelated sites of inflammation
- Not sensitive enough to discriminate intermediate stages
- Dependent on clearance rate and influenced by impaired biliary function and renal excretion
- None of the biomarkers have a high degree of accuracy
- Most of the biomarkers need further validation

## Diagnosis & assessment of liver fibrosis

**Figure 2.**
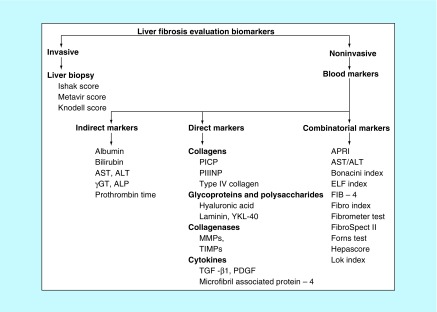
**Algorithm of liver fibrosis markers.** γGT: Gamma glutamyl transferase; ALP: Alkaline phosphatase; ALT: Alanine aminotransferase; APRI: AST to platelet ratio index; AST: Aspartate aminotransferase; ELF: Enhanced liver fibrosis; FIB-4: Fibrosis-4; MMP: Matrix metalloproteinase; PICP: Procollagen I carboxy peptide; PIIICP: Procollagen III amino peptide; TIMP: Tissue inhibitor of metalloproteinase.

Accurate assessment of the extent of liver fibrosis is essential for clinical management so as to predict prognosis and therapeutic decision in patients with liver fibrosis (Figure 2).

## Liver biopsy

**Figure 3.**
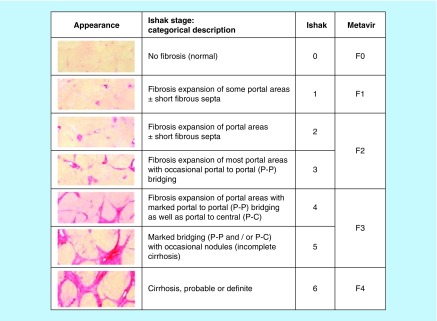
**Histological scoring system for liver fibrosis.** Reproduced with permission from [10].

Despite development of potential diagnostic tests for the past 50 years, liver biopsy is considered as gold standard method to classify liver fibrosis and provides useful information about diagnosis and also other damaging process viz., necrosis, inflammation and steatosis [9]. Three of the widely used methods to assess histological fibrosis are: Ishak score, Metavir score and Desmet/Scheuer staging system (Figure 3) [10]. Each scoring system relies on progressive development of periportal fibrosis followed by septal fibrosis and finally nodule formation [11].

Limitation of liver biopsy is highly invasive. Moreover, poor sample quality and tissue size make biopsy nonreproducible and depend on the experience of pathologist which leads to interobserver variations. Risk allied for liver biopsy range from pain (84%) and hypertension, bleeding (0.5%) and damage to biliary system with approximately 0.01% mortality rate [12]. These limitations of liver biopsy have given urgency for development of noninvasive diagnostic procedure for liver fibrosis. An ideal biomarker should be organ specific, sensitive to indicate active damage, easily accessible in peripheral tissue and cost effective [13]. Advantages of biomarkers over liver biopsy are that their estimations in serum are by minimal invasive procedure. Further advantages are easy applicability, interlaboratory reproducibility and broad availability.

Serum biomarkers for liver fibrosis are classified into two categories [14]:

- Direct markers: which reflects ECM turnover
- Indirect markers: molecules released into blood which reflect alterations of hepatic function

## Direct markers of liver fibrosis

Direct markers are directly involved in deposition and removal of ECM produced by HSC and other hepatic cells. Serum levels of these markers are elevated with progressing fibrosis and have a tendency to decrease with response to treatment [15]. Assessment of these markers may be useful for bringing about effective treatment, but they are neither organ specific nor readily available. Direct markers of liver fibrosis are classified according to their molecular structure (Table 1) [16].

**Table 1.** **Classification of direct markers for liver fibrosis according to structure.**

**Collagens** – PICP – PIIINP – Type IV collagen	**Collagenases and their inhibitors** – MMPs – TIMPs

**Glycoproteins and polysaccharides** – Hyaluronic acid – Laminin – YKL-40	**Cytokines and proteomic markers** – TGF-β1 – PDGF – Microfibril associated protein-4

MMP: Matrix metalloproteinase; PICP: Procollagen I carboxy peptide; PIIINP: Procollagen III amino peptide; TIMP: Tissue inhibitors of metalloproteinase.

Data taken from [16].

## Collagens

### Procollagen I carboxy peptide & procollagen III amino peptide

During synthesis of collagen, procollagen undergoes enzymatic cleavage at carboxy and amino terminal ends by procollagen C-peptidase and procollagen N-peptidase and peptides are released into serum whose estimations can be used to assess matrix deposition [17]. Fibril-forming type I collagen is profuse in healthy liver. During fibrogenesis, type I collagen will be increased up to eightfold [18]. Serum estimations can give an indication regarding the severity of disease. Type III collagen, a fibril-forming collagen is an important component of connective tissue. Concentrations of procollagen III amino peptide (PIIINP) in basal membrane are greater during hepatic fibrosis due to chronic liver injury. PIIINP will be correlated with aminotransferase levels in acute hepatitis which reflects degree of fibrosis [18,19].

Relatively low sensitivity and specificity (78 and 81%) of these markers have limited their clinical use. There is no correlation between procollagen I carboxy peptide and PIIINP serum levels with histological grading of liver fibrosis. Hence, these are not reliable to establish fibrosis grading [19,20].

### Type IV collagen

Type IV collagen is a crucial component of hepatic ECM which is deposited integrally in matrix. Serum estimation of type IV collagen is a sign of direct degradation and has positive correlation with grade of liver fibrosis. Combinatorial use of type IV collagen with PIIINP has a sensitivity and specificity of 88% [19,21].

## Glycoproteins & polysaccharides

### Hyaluronic acid

HA is a glycosaminoglycan synthesized by HSCs and is the main component of ECM. In normal liver, HA uptake and degradation take place in hepato sinusoidal endothelial cells. Increased concentrations in serum are attributable to increased production and decreased hepatic elimination or both [22]. Serum HA levels are related to stage of fibrosis and degree of necroinflammation. High levels have been detected in liver fibrosis with varied etiology [23]. HA has sensitivity and specificity of 88–95% and 86–100%, respectively, in liver fibrosis especially nonalcoholic fatty liver diseases, but positive and negative predictive value of HA has been reported as 61% and 98–100%, respectively [18].

### Laminin

Laminin is noncollagenous glycoprotein deposited in basal membrane of liver, synthesized by HSCs. In liver fibrosis, laminin increases around the vessels, in perisinusoidal space and portal triad. Serum laminin levels are elevated in liver fibrosis irrespective of etiology and have a correlation with severity of fibrosis and liver inflammation [24]. Laminin cut-off concentration at 1.45 U/ml has sensitivity and specificity of 87 and 74%, respectively, with positive-predictive value of 77% and negative-predictive value of 85%. Estimations of serum HA and laminin have good prognostic value for liver fibrosis complications [25].

### YKL-40

YKL-40 (chondrex, human cartilage glycoprotein-39) is a glycoprotein. YKL-40 mRNA is strongly expressed by liver [26]. It can be used as a marker to assess liver fibrosis and helps distinguish between mild stage and extensive stage of liver fibrosis and has positive-predictive value of 80%. Between HA and YKL-40, HA is a better predictive marker for liver fibrosis [27].

## Collagenases & their inhibitors

### MMPs & TIMPs

Degradation of ECM of liver is due to activity of MMP. Three MMPs are expressed in humans viz., MMP-1 (collagenases), MMP-2 (gelatinase A) and MMP-9 (gelatinase B) [28]. These enzymes are synthesized intracellularly and secreted as zymogens. MMPs are activated by membrane-type MMP and inhibited by tissue inhibitors of metalloproteinases (TIMPs) [15]. In liver fibrosis, there will be inverse correlation between levels of MMP-1 and histological severity [29]. MMP-2 secreted from hepatic stellate cells in liver disease has high diagnostic accuracy of 92% to detect liver fibrosis. There will be a 2.4-fold increase in the levels of MMP-2 in fibrotic patients when compared with controls [30]. MMP-9 from Kupffer cells has negative correlation with histological severity [31].

ECM degradation by MMPs is inhibited by TIMPs, which affect MMPs function. TIMP-1 will interact with almost all the 3MMPs where as TIMP-2 specifically interacts with MMP-2. With progression of liver disease, serum levels of TIMPs will increase. MMP-1/TIMP-1 ratio is useful for the diagnosis of hepatic fibrosis and correlates with degree of portal inflammation [32].

## Cytokines & proteomic markers

### TGF-α & TGF-β1

In liver fibrosis, TGF-α enhances proliferation of HSCs and correlates well with progression of the disease [33,34]. Homodimetric polypeptide, TGF-β1, secreted in an inactive form, has pleiotropic effect through membrane receptors. TGF-β1 stimulates production of ECM by HSCs and inhibits hepatocyte growth and proliferation in liver fibrosis [35]. High levels of TGF-β1 correlate with progression of hepatic fibrosis. TGF-β1 cut-off value of less than 75 ng/ml is an indicator of stable disease. Limitation of levels of TGF-β1 is due to contamination of sample by platelet TGF-β [36].

### PDGF-BB

PDGF-BB is expressed by platelets, fibroblasts, endothelial cells, mast cells and macrophages [37]. It is the main subunit which stimulates HSC proliferation and migration. Serum levels of PDGF-BB have correlation with severity of hepatic fibrosis. In early studies by Pinzani *et al.* and Ikura *et al.*, PDGF-BB mRNA expression was found to be markedly elevated in chronic liver disease [38,39]. Recent studies by Yoshida *et al.* and Jiyuan *et al.* showed decreased serum levels of PDGF-BB in liver fibrosis [37,40].

### Microfibrillar-associated protein 4

Microfibrillar-associated protein 4 present in ECM including elastin and collagen is a disulfide-linked dimer that forms higher oligomeric structure [41]. In its C-terminal end, MFAP4 has fibrinogen like domain and in the N-terminal end an integrin binding motif is present [42]. Recent studies suggest that MPAF4 has a sensitivity of 91.6% and a specificity of 95.6%. MPAF4 is an ideal serum marker among liver-specific proteins [43].

### Cytokeratin-18 fragments

The major intermediate filament present in hepatocyte are cytokeratin-18 fragments (CK18). Caspase-induced apoptosis takes place by cleavage of CK18 in different positions and results in the formation of CK18 fragments [44]. According to Yilmaz *et al.*, levels of M30 antigen (a neoepitope in CK18) and M65 (cytosolic pool of CK18) can distinguish between advanced fibrosis and early-stage fibrosis [45,46].

Although a single direct marker may serve as an indicator of disease severity, there is growing consensus that combination of multiple markers as an integrated panel will enhance the performance characteristics in terms of specificity and sensitivity.

According to Oberti *et al.*, best diagnostic accuracy was found for HA (86%), laminin (81%), PIIINP (74%) and TGF-β (67%) [15]. But in this study, the diagnostic advantages over nonspecific markers like prothrombin index, gamma glutamyl transferase (yGT), and α2 macroglobulin were not reported [15]. Murawaki *et al.* inferred that HA and MMP-2 are useful for diagnosing stages of fibrosis, but cannot replace liver biopsy as there is an overlap among stages and grades in liver fibrosis [47]. The European Liver Fibrosis study compared the diagnostic performance of HA, PIIINP and TIMP-1 with liver biopsy with threshold sensitivity greater than 90% and specificity greater than 90% can detect liver fibrosis [48]. Patel *et al.* observed that diagnostic value of HA, TIMP-1 and α2-macroglobulin can differentiate chronic hepatitis C patients with moderate/severe fibrosis from those with no or mild fibrosis (Table 2) [49,50].

**Table 2.** **Area under receiver's operating curve for direct markers in various etiology of liver fibrosis.**

**Marker**	**Liver disease evaluated by biochemical marker**				**AUROC for advanced fibrosis**

	***CHC***	***CHB***	***NAFLD***	***ALD***	
PICP	NA	–	–	NA	NA

PIIINP	0.69–0.78	–	NA	0.67–0.87	0.67–0.87

Type IV collagen	0.73–0.83	–	0.82	NA	0.58–0.83

HA	0.82–0.92	0.98	0.97	0.69–0.93	0.69–0.98

Laminin	0.54–0.82	–	NA	NA	0.46–0.82

YKL-40	0.7–0.81	–	NA	NA	0.7–0.81

MMP-2	0.59	–	–	–	0.59

ALD: Alcoholic liver disease; AUROC: Area under receiver's operating curve; CHB: Chronic hepatitis B; CHC: Chronic hepatitis C; HA: Hyaluronic acid; MMP: Matrix metallo proteinase; NA: AUROC is not available; NAFLD: Nonalcoholic fatty liver disease; PICP: Procollagen I carboxy peptide; PIIINP: Procollagen III amino peptide.

Data taken from [50].

### Indirect markers of liver fibrosis

Indirect markers reflect alteration in hepatic function. These markers are useful in diagnosing, evaluating severity, monitoring therapy and also assessing the prognosis of liver diseases. These include measurement of activity of enzymes viz., aminotransferases, alkaline phosphatase (ALP) and γ-glutamyl transferase (γGT), and estimations of bilirubin and albumin in blood [51]. These are the markers for liver injury, not for liver function and should be referred as liver chemistries or liver tests [52].

### Aminotransferases

Liver disease is most important cause of increased transaminase activity in serum. Serum activities of aspartate aminotransferase (AST; EC 2.6.1.1) and alanine aminotransferase (ALT; EC 2.6.1.2) are elevated when disease processes affect liver cell integrity. Between these two, ALT is more specific enzyme for liver insult. Alterations of ALT activity persist longer than AST activity. Activities of both enzymes may reach as high as 100-times upper reference limit in liver diseases. Peak activities bear no relationship to prognosis and may fall with worsening of patient's condition [53]. AST/ALT ratio >1 is a prediction of cirrhosis, and has sensitivity and specificity of 81.3 and 55.3%, respectively. In some etiologies of chronic hepatitis, the ratio is ≤1, whereas ratio >2 suggests alcoholic hepatitis [54].

### Alkaline phosphatase (EC 3.1.3.1)

Zinc metalloproteinase enzyme, ALP, catalyzes the hydrolysis of phosphate esters at an alkaline pH. The response of liver to any form of biliary tree obstruction induces the synthesis of ALP from canalicular membrane of hepatocytes [52]. Thus newly formed enzyme enters the circulation to increase the enzyme activity in serum. Elevation tends to be more notable in extra hepatic obstruction than in intrahepatic obstruction. Serum enzyme activities may reach 10- to 12-times the upper reference limit. Liver diseases that principally affect parenchymal cells such as infectious hepatitis typically show only moderate increase or even normal serum ALP activity. Increase may also be seen as a consequence of response to drug therapy [55].

### γGT (EC 2.3.2.2)

Elevated activities of γGT are found in serum of alcoholic hepatitis patients. Moderate elevations occur in infectious hepatitis. Increased concentrations of enzyme are also found in serum of subjects receiving anticonvulsant drugs (phenytoin and phenobarbital). γGT is a sensitive indicator and elevated in most of the subjects with liver disease regardless of cause, but its efficacy is limited due to lack of specificity [52,55].

### Albumin

Liver has synthesizing capacity to maintain albumin concentrations until parenchymal damage is more than 50%. Plasma albumin measurements are useful in assessing chronicity and severity of the disease. However, its utility for this purpose is limited, as plasma albumin concentration is also decreased in acute kidney disease [55].

### Bilirubin

Sequential measurement of bilirubin is supportive in assessing the severity of liver damage due to different etiology. In acute hepatitis, serum bilirubin peaks later than enzymes but remains elevated for longer time [52,55].

### Prothrombin time

Serial PT measurements can be used to differentiate between cholestasis and severe hepatocellular diseases. In severe hepatocellular damage, PT remains elevated for a longer time. Cholestasis will cause a decrease in PT as a result of malabsorption of vitamin K [15,55].

### Combinatorial use of biomarkers

Combination of different markers can improve sensitivity and specificity of these tests (Table 3) [50].

**Table 3.** **Area under receiver's operating curve for indirect marker panel in various etiology of liver fibrosis.**

**Marker**	**Liver disease evaluated by biochemical marker**				**AUROC for advanced fibrosis**

	***CHC***	***CHB***	***NAFLD***	***ALD***	
AST/ALT ratio	0.54–0.71	NA	0.74–0.83	NA	0.54–0.83

APRI	0.65–0.87	0.67–0.72	0.56–0.86	–	0.56–0.87

FibroTest	0.72–0.87	0.76–0.85	0.82–0.89	0.83–0.91	0.72–0.87

Fibro index	0.8–0.83	NA	–	–	0.82

Frons index	0.78–0.86	NA	–	–	0.78–0.86

ALD: Alcoholic liver disease; ALT: Alanine aminotransferase; APRI: AST to platelet ratio index; AST: Aspartate aminotransferase; AUROC: Area under receiver's operating curve; CHB: Chronic hepatitis B; CHC: Chronic hepatitis C; NA: AUROC is not available; NAFLD: Nonalcoholic fatty liver disease.

Data taken from [50].

### AST/platelet ratio (APRI)



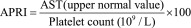



Wai *et al.* developed 'AST to platelet ratio index' (APRI). APRI more than 1.5 has area under receiver's operating curve (AUROC) of 80% and 89% for advanced fibrosis F3–F4 and cirrhosis, respectively [56]. According to Snyder *et al.*, APRI cut-off of 0.42 or less has high diagnostic accuracy with a negative predictive value (NPV) of 95% [57]. In autoimmune hepatitis, Loaeza Del Castillo *et al.* showed that APRI does not have any diagnostic value in assessing fibrosis [58]. Lok *et al.* enhanced diagnostic accuracy of APRI in incorporating ALT and international normalized ratio in assessing the progression of fibrosis in postliver transplant patients [59].

### Bonacini index



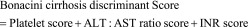



Bonacini *et al.* developed a discriminant score (Table 4) [60] for diagnosis of advanced fibrosis and cirrhosis by taking three parameters: platelets, ALT/AST ratio and PT which have positive correlation with histological scores and have 98% specificity but 46% sensitivity [60].

**Table 4.** **Bonacini cirrhosis discriminant parameters score.**

**Score**	**Platelets (10^3^/μl)**	**ALT:AST ratio**	**INR**
0	>340	>1.7	<1.1

1	280–340	1.2–1.7	1.1–1.4

2	220–279	0.6–1.19	>1.4

3	160–219	<0.9	–

4	100–159	–	–

5	40–99	–	–

6	<40	–	–

ALT: Alanine aminotransferase; AST: Aspartate aminotransferase; INR: International normalized ratio.

Data taken from [60].

### FIB-4 score



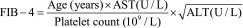



Sterling *et al.* developed a score to assess fibrosis in HIV/HCV coinfected patients and successfully classified 87% of patients at a cut-off of 3.25 with an AUROC of 76% [61]. Further validation of this score showed AUROCs of 85 and 81% for monoinfected HCV and HBV patients, respectively [62,63].

### Fibro index



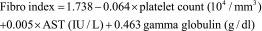



Koda *et al.* developed score from platelet count, AST and γGT to assess fibrosis [63]. A cut-off of 2.25, was correlated with F2–F3 fibrosis and has 90% NPV [64]. However, further validation showed this score has less diagnostic accuracy [65].

### FibroTest



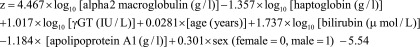



FibroTest (Fibro Sure in USA) was patented since 2001 by APHP (Assistance publique - Hopitaux de Paris), the Parisian public hospital system. It is the most validated test and is based on age, gender, serum haptoglobin, α2 macroglobulin, apolipoprotein A1, γGT and bilirubin. However, it is less significant in detection of intermediate stages of fibrosis (Table 5) [66]. Poynard *et al.* established high accuracy of FibroTest in steatohepatitis with AUC of 85% [67].

**Table 5.** **Conversion between FibroTest and fibrosis stages.**

**FibroTest**	**METAVIR score**	**Knodell score**	**Ishak score**
0.75–1.00	F4	F4	F6

0.73–0.74	F3–F4	F3–F4	F5

0.59–0.72	F3	F3	F4

0.49–0.58	F2	F1–F3	F3

0.32–0.48	F1–F2	F1	F2–F3

0.28–0.31	F1	F1	F2

0.22–0.27	F0–F1	F0–F1	F1

0.00–0.21	F0	F0	F0

Conversion between FibroTest and fibrosis stages using METAVIR, Knodell and Ishak fibrosis scoring systems.

Data taken from [66].

### Forns index



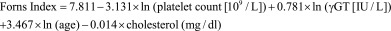



In 2002, Forns *et al.* developed this score by calculating age, platelet count, serum cholesterol and γGT which can differentiate mild fibrosis with advanced fibrosis at a cut-off value of 6.9 [68]. Further validation of this index showed sensitivity of 94%, specificity of 51% with AUROC ranging from 81 to 86% [69].

### PGA index

- PT (% of control): ≥80 = 0; 70–79 = 1; 60–69 = 2; 50–59 = 3; <50 = 4
- γGT (IU/l): <20 = 0; 20–49 = 1; 50–99 = 2; 100–199 = 3; ≥200 = 4
- Apolipoprotein A1 (mg/dl): ≥200 = 0; 175–199 = 1; 150–174 = 2; 125–149 = 3; <125 = 4
- α2 macroglobulin (g/l): <1.25 = 0; 1.25–1.74 = 1; 1.75–2.24 = 2; 2.25–2.74 = 3; ≥2.75 = 4

PGAA index is the sum of the above.

Poynard *et al.* anticipated PGA index in combination with γGT, prothrombin index and apolipoprotein A to assess alcoholic liver disease [70]. The accuracy of this index has been increased from 65 to 70% by addition of α2 macroglobulin (PGAA) [71].

Calculating such a score greatly improves sensitivity and specificity and can avoid limitations of individual markers. Combinations of direct and indirect markers may increase diagnostic accuracy, but has not been implemented in clinical practice (Table 6) [72]. Scores may give clear positive or negative prediction only at early stages of fibrosis. In acute hepatic injury, there will be false positive results in scores such as APRI, Forns index and FIB-4. In hemolytic and hyper bilirubinemia, false positive results may be possible for FibroTest [21]. According to WHO 2015 report, APRI and FibroTest are preferred noninvasive tests to assess the presence of cirrhosis caused by hepatitis B [73]. APRI has low performance when compared with FIB-4 and FibroTest in liver disease caused by hepatitis B and hepatitis C [74]. FIB-4 cut-offs were initially validated only for F3 and F4, and need specific validation before comparing with FibroTest and APRI [61].

**Table 6.** **Main scoring system for liver fibrosis with sensitivity and specificity.**

**Test**	**Parameters**	**Sensitivity (%)**	**Specificity (%)**
APRI	AST/platelet count	57	93

AST/ALT	AST/ALT	51	71

Bonacini index	ALT/AST, INR, platelet count	46	98

ELF index	Age, HA, PIIINP and TIMP-1	90	69

FIB-4	Platelet count, AST, ALT and age	65	97

Fibro index	Platelet count, AST and γ-globulin	35	97

Fibrometer test	Platelet count, INR, AST, α2 macroglobulin, HA, urea and age	80	84

FibroSpect II	HA, TIMP-II and α2 macroglobulin	76	73

Forns test	Age, platelet count, γGT and cholesterol	30	95

Globulin–albumin ratio	Globulin and albumin	43	98

GUCI	Platelet count, AST and INR	80	78

Hepascore	Age, gender, bilirubin, γGT, HA and α2 macroglobulin	84	71

Lok index	Platelet count, AST, ALT and INR	68	72

γGT: Gamma glutamyl transferase; ALT: Alanine aminotransferase; APRI: AST to platelet ratio index; AST: Aspartate aminotransferase; ELF: Enhanced liver fibrosis; FIB-4: Fibrosis-4; GUCI: Goteborg University cirrhosis index; HA: Hyaluronic acid; INR: International normalized ratio; PIIINP: Procollagen III amino peptide; TIMP-1: Tissue inhibitor of metalloproteinase I; TIMP-II: Tissue inhibitor of metalloproteinase II.

Data taken from [72].

### Evolving biomarker candidates for liver fibrosis

α-smooth muscle actin is an isoform of actin expressed from myofibroblasts which plays an important role in fibrogenesis. Active myofibroblasts proliferate and synthesize large amounts of extracellular components. Expression of α-smooth muscle actin correlates with activation of myofibroblasts, and is a reliable marker for HSCs activation [75–77]. Maieron *et al.* identified Von Willebrand factor as a new biomarker for chronic liver diseases; further established VITRO score (Von Willebrand factor-Ag/PLT) to evaluate stages of liver fibrosis in chronic hepatitis C patients [78].

White *et al.* and Gangadharan *et al.* discovered protein marker candidates: apolipoprotein AIV, lipid transfer inhibitory protein, complement C3, apolipoprotein L1, apolipoprotein J and corticosteroid-binding protein for liver fibrosis by proteomic approach in various studies [79]. Zhiyun *et al.* observed the alterations in concentrations of Kallistatin depending on the degree of severity of disease and also have documented that Kallistatin levels in serum vary in different liver diseases (fibrosis, cirrhosis and hepatocellular carcinoma) [80]. Irvine *et al.* identified 17 analytes with differential expression between patients with no advanced fibrosis and patients with advanced fibrosis which have the potential to enhance the diagnostic accuracy. Data suggest that MMP7 is a valuable indicator of advanced fibrosis [81].

Hu *et al.* discovered eight marker candidates viz., malic acid, oxidized glutathione, γ-glutamyl-cysteinyl-glycine, ATP, phenylalanine, AMP, nitrotyrosine and tryptophan by metabolomic approach [82]. Zeng *et al.* showed the use of ceruloplasmin to identify various liver fibrosis stages via AUROC values [83]. They further developed a model in combination with ceruloplasmin and γGT, which has sensitivity and specificity of 84 and 83.1%, respectively [83]. Newly discovered candidate markers may have vital responsibility for assessment of chronic liver injury which needs further evaluation. Statistical comparison should be made with established biomarkers and panels.

### Genetic markers for liver fibrosis

Genetics of progression in liver fibrosis is multifactorial (genes, environmental factors and cell types) and highly complex. Hall *et al.* identified seven  genomic loci on chromosomes 4, 5, 7, 12 and 17 which influences fibrosis phenotypes based on quantitative trait locus analysis [84]. Aravinthan *et al.* investigated the relationship between the variants of *CDKN1A* in different population and concluded that *CDKN1A* variant rs762623 related to the development but not the progression of liver disease in nonalcoholic fatty liver disease [85]. Lopez-Rodriguez *et al.* identified seven single nucleotide polymorphisms located in *IL-28B* (rs12979860), *JAK1* (rs11576173 and rs1497056), *TYK2* (rs280519), *OAS1* (rs2057778), *SOCS1* (rs33932899) and *RNASEL* (rs3738579) genes in severe necroinflammatory activity grade of chronic hepatitis C patients [86]. The genotypes of IL-10–1082G/A and TNF-α 308G/A expressed elevated levels of inflammatory cytokines in nonalcoholic steatohepatitis’ patients can signify changes in liver functions, disease severity and to forecast the risk for progression [87].

Epigenetic mechanisms (DNA methylation, histone modification and noncoding RNA mediated gene silencing) regulate chromatin structure, modification and initiation of transcription are involved in fibrogenesis of liver. Epigenome is influenced by age, gender, environment and underlying genome through presence of single nucleotide polymorphisms [88,89]. Abnormal DNA methylation patterns are associated with inappropriate gene repression in liver fibrosis. Differential DNA methylation at peroxisome proliferator-activated receptor-γ promoter in cell free DNA may distinguish mild with severe liver fibrosis [90].

Small, noncoding micro RNAs (miRNAs) regulate gene expression by binding to mRNA and control diverse biological functions viz., apoptosis, cell proliferation and differentiation [91,92]. Alterations of intracellular miRNAs play an important role in pathophysiology of chronic liver disease with different etiology. Normal liver homoeostasis requires miR-122 which regulates genes that are involved in hepatic cholesterol and lipid metabolism [93]. After chronic liver injury, HSCs’ proliferation and differentiation into myofibroblast-like cells are regulated by mi-R221. miR-9, miR-21 and miR-188 regulate activation of myofibroblast, synthesis of extracellular proteins and collagen deposition. The broad variety of miRNAs which are involved in liver fibrosis and enters into systemic circulation can serve as potential biomarkers. Studies show that identification of circulating miRNA expression profiles are distinct between liver diseases with varied etiology (Table 7) [94].

**Table 7.** **Circulating miRNA signatures in liver disease.**

**Etiology**	**miRNA signature**	**Expression**
ALD	miR-122 (acute alcohol, microsteatosis) miR-122 and miR-155 (chronic alcohol, macrosteatosis)	Increases Increases

NAFLD/NASH	miR-122, miR34a and miR-192	Increases

HCV	miR-122, miR-34a, miR-155, miR-125b, miR-146a and miR-21	Increases

HBV	miR-192 and miR-122	Increases

Liver fibrosis/ cirrhosis	miR-29 and miR-652 miR-513-3p and miR-571	Decreases Increases

HCC	miR-21, miR-16, miR-199a, miR-122, miR-223 and miR-885-5p	Increases

Drug overdose	miR-122 and miR-192	Increases

ALD: Alcoholic liver disease; HBV: Hepatitis B virus; HCC: Hepatocellular carcinoma; HCV: Hepatitis C virus; NAFLD: Nonalcoholic fatty liver disease; NASH: Nonalcoholic steatohepatitis.

Reproduced with permission from [94].

## Future perspective

Existing biomarkers for liver fibrosis in clinical practice have narrow applicability due to lack of specificity (predict etiology) and lack of sensitivity (distinguish intermediate stages). An ideal biomarker would give insights for diagnosis, monitor the activity of disease and assess therapeutic response. The determination of biomarkers could be an easy, noninvasive and inexpensive method to monitor the progression of liver fibrosis. This leads to urgency in the progression of biomarker discovery for liver fibrosis and hepatotoxicity with the help of advances in -omics approach. Discovery of biomarker candidates should be a simplified, unbiased, semi-quantitative binary comparison between diseased and normal. During discovery phase, the variables (study design, preanalytical and analytical) which affect precision should be minimized. Newly identified biomarker candidates need validation in terms of performance characteristics. Biomarker validation (analytical validation, clinical validation and clinical utility) links biomarker with biological process and clinical end point and is necessary for fit-for-purpose which helps research data for better patient care.

Analytical validation is to develop optimized assay which has consistency to measure the specific biomarker. Considerable care should be taken during the process in terms of preanalytical variation, interfering substance, indicators of accuracy, precision, analytical measurement range and proficiency testing procedures. Clinical validation gives diagnostic accuracy and discriminates between those with and without disease in terms of sensitivity, specificity, likelihood ratio and receiver operating characteristics curve. Likelihood ratio and receiver operating characteristics curve are derived from sensitivity and specificity values of a biomarker. Clinical utility of a biomarker is evaluated in a series of human population in terms of performance characteristics which needs stratified and sub population studies across geographical setting. To overcome the pitfalls in translation from biomarker discovery to clinical utility, there is a need for definite study design in selection of patients, proper biomarker validation and robustness in analytical techniques.

**Executive summary**

**Liver fibrosis**

- Development of liver fibrosis is a pathological condition caused by varied etiology and is associated with significant morbidity and mortality.

**Liver biopsy**

- An invasive liver biopsy is still considered as a gold standard diagnostic tool for liver fibrosis associated with pain and complications.

**Direct & indirect biochemical markers**

- No doubt extracellular matrix markers and cytokines have diagnostic value, but they do not have much significance compared with routine biomarkers.
- Moreover, they are not organ specific and may also correlate with diseases in other organs.

**Combinatorial biochemical markers**

- Scoring system for diagnosis and prognosis of liver fibrosis plays a role only after pathological outcome.

**Future perspectives for liver fibrosis markers**

- Considering these limitations, successful management of liver fibrosis needs more reliable biomarkers which are specific to liver.
- In this regard, newly discovered biomarkers may have vital responsibility for assessment of chronic liver injury which needs further evaluation and statistical comparison with established biomarkers and panels.

**Conclusion**

- In present scenario, there is a need for extensive research to establish an accurate, precise, organ specific and sensitive noninvasive biomarker for early diagnosis, management and therapeutic monitoring of liver fibrosis.
